# Photogenerated Intrinsic Free Carriers in Small-molecule Organic Semiconductors Visualized by Ultrafast Spectroscopy

**DOI:** 10.1038/srep17076

**Published:** 2015-11-27

**Authors:** Xiaochuan He, Gangbei Zhu, Jianbing Yang, Hao Chang, Qingyu Meng, Hongwu Zhao, Xin Zhou, Shuai Yue, Zhuan Wang, Jinan Shi, Lin Gu, Donghang Yan, Yuxiang Weng

**Affiliations:** 1 Laboratory of Soft Matter Physics, Institute of Physics, Chinese Academy of Sciences (CAS), Beijing 100190, China; 2 State Key Laboratory of Polymer Physics and Chemistry, Changchun Institute of Applied Chemistry, CAS, Changchun 130022, China; 3 Laboratory of Solid State Quantum Information and Quantum Computation, Institute of Physics, CAS, Beijing 100190, China; 4 State Key Laboratory of Catalysis, Dalian Institute of Chemical Physics, CAS, Dalian 116023, China; 5 Laboratory of Advanced Materials & Electron Microscopy, Institute of Physics, CAS, Beijing 100190, China

## Abstract

Confirmation of direct photogeneration of intrinsic delocalized free carriers in small-molecule organic semiconductors has been a long-sought but unsolved issue, which is of fundamental significance to its application in photo-electric devices. Although the excitonic description of photoexcitation in these materials has been widely accepted, this concept is challenged by recently reported phenomena. Here we report observation of direct delocalized free carrier generation upon interband photoexcitation in highly crystalline zinc phthalocyanine films prepared by the weak epitaxy growth method using ultrafast spectroscopy. Transient absorption spectra spanning the visible to mid-infrared region revealed the existence of short-lived free electrons and holes with a diffusion length estimated to cross at least 11 molecules along the π−π stacking direction that subsequently localize to form charge transfer excitons. The interband transition was evidenced by ultraviolet-visible absorption, photoluminescence and electroluminescence spectroscopy. Our results suggest that delocalized free carriers photogeneration can also be achieved in organic semiconductors when the molecules are packed properly.

Organic semiconductors, such as π-conjugated polymers and small-molecule crystals, are promising materials for future applications ranging from flexible electronics to photovoltaics^1^,^2^. However, in contrast to the widely-used inorganic semiconductor, theoretical description of organic semiconductors is still controversial in spite of decades of intense research since organic semiconductors stand in an intricate region between the molecular limit (exciton) and extended band (free carrier) descriptions^3^,^4^. Rather than direct generation of free electrons and holes by interband photoexcitation in conventional inorganic semiconductors, the optical excitation in organic semiconductors is generally believed to be excitonic leading to the formation of spatially localized bound electron-hole pairs (excitons). Such a discrepancy is caused by: (i) far weaker intermolecular electronic coupling and (ii) much lower static dielectric constant which results in a poor screening effect for Columbic interactions in organic semiconductors.

Despite of the disadvantages described above, progresses towards ultrafast spectroscopic characterizations of neat polymer films have evidenced simultaneous ultrafast generation of excitons and polarons upon photoexcitation^5^,^6^,^7^. Still, it is noteworthy to point out that, while acknowledging the great similarities in electronic and optical properties of polymer and small-molecule organic thin films, an important difference exists in that the “soft” chains of polymers tend to localize the charge carriers due to polaronic relaxation, whereas small-molecule crystals possess rigid lattice structures leading to lower reorganization energies and more delocalized charge wave functions^8^. It has been shown that intrinsic charge carrier transport in some sufficiently pure and perfect low molecular weight organic crystals can safely be described within the framework of coherent Bloch-type band states as long as temperature is sufficiently low^9^. Angular photoemission spectroscopy studies of pentacene crystal and thin film have shown that the highest occupied orbitals exhibit band-like structures^4^,^10^. Coherent band-like carrier transport has been validated by experimental observations, such as mobility increasing with decreasing temperature^11^, free hole absorption based on the Drude model^12^ and Hall effects^13^,^14^, in small-molecular organic thin films, where the carriers were accumulated through electrode injection or heterojunction effect. Furthermore, facts from the transient photoconductivity^15^ and ultrafast carrier dynamics by optical pump-terahertz probe techniques^16^ of single crystals of pentacene, rubrene, etc., point to the existence of free carriers in them after primary photoexcitation. Nevertheless, the prevailing consensus addressing photoexcitation mechanism in small-molecule semiconductors remains excitonic, and the description of the charge generation process in small-molecule based photovoltaic devices is still within the framework of exciton dissociation^17^,^18^. The exact nature of charge carrier photogeneration and evolution in small-molecule organic semiconductors remain poorly understood due to the difficulties in accessing the intrinsic properties that are often masked by impurities, contact effects, or grain boundaries^12^, and unambiguous evidences of direct photogeneration of delocalized free carriers in organic semiconductors are still lacking.

To address the above issue, noncontact steady-state and ultrafast time-resolved spectroscopic methods were employed to characterize the intrinsic photoexcitation properties of a typical highly crystalline small-molecule organic semiconductor, i.e. zinc phthalocyanine (ZnPc) films prepared by weak epitaxy growth (WEG) method. Our results clearly demonstrate the existence of directly photogenerated delocalized free carriers originated from interband transition within these highly crystalline films. The free carrier generation and subsequent localization processes forming charge transfer (CT) excitons were visualized with ultrafast time-resolved spectroscopy by observation of the transient absorption signatures for fully oxidized ZnPc cations, free electrons and partially oxidized ZnPc cations.

We have noted that there have been a number of works reporting ultrafast time-resolved THz spectroscopic study of ZnPc/C_60_ films of blending and multilayer structures as well as vacuum deposited films of ZnPc only. Interestingly, while time-resolved THz spectroscopy measurements of the ZnPc/C_60_ blending and multilayer films show significant THz absorption attributed to charge generation by the dissociation of excitons at the ZnPc/C_60_ heterojunction interface, the time-resolved THz signal for vacuum deposited ZnPc films are negligible, which suggests negligible photogenerated free carriers in vacuum deposited ZnPc films^19^,^20^,^21^.

## Results and Discussions

### Steady-state spectroscopic characterization

**Figure 1:**
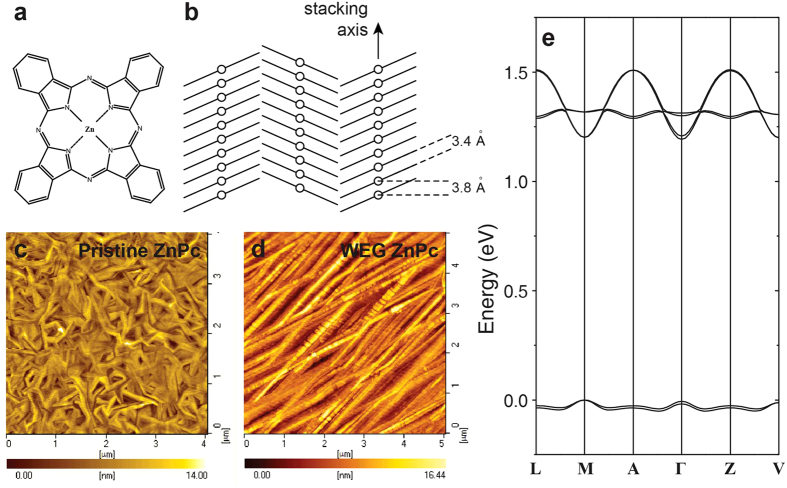
Physical and electronic structures of ZnPc films. (**a**) The molecular structure of ZnPc. (**b**) Herringbone molecular arrangement in the α-ZnPc crystal. (**c,d**) AFM images of pristine ZnPc and WEG ZnPc films respectively. (**e**) Calculated band structure of the α-ZnPc crystal (see Supplementary Fig. 1 for the Brillouin zone path).

Two kinds of ZnPc films were prepared (see Supplementary Information), one by direct vacuum deposition (known as pristine ZnPc films), while the other by applying WEG method in vacuum deposition (referred to as WEG ZnPc films). Both films were determined to be in the α-phase by X-ray diffraction (XRD) and selected area electron diffraction (SAED)^22^. However, the XRD diffraction peaks of WEG ZnPc films are much sharper, indicating a much higher crystallinity, and show a small shift with respect to those of pristine ZnPc films, equivalent to a shrink of 0.1 Å in the corresponding lattice spacing, revealing a tighter packing pattern for WEG ZnPc films. Furthermore, WEG ZnPc films have been proved to exhibit a low deep trap state density and a field-effect hole mobility as high as 0.32 cm^2^V^–1^s^–1^, both of which improve by one order of magnitude comparing to that of pristine ZnPc films^22^,^23^. Figure 1a,b present the molecular structure of ZnPc and the molecule stacking pattern in the α-ZnPc crystal. The molecules are cofacially stacked with a spacing of ~3.4 Å^24^, which is slightly larger than the shortest achieved π-π stacking distance in an organic semiconductor crystal lattice (3.08 Å)^25^. The stacked molecule arrays are arranged in a herring-bone pattern showing two inequivalent molecules in a unit cell (also see Supplementary Fig. 1). The atomic force microscopy (AFM) images of pristine ZnPc and WEG ZnPc films are compared (Fig. 1c,d). In contrast to that of the pristine film, it could be seen that highly oriented and continuous crystal grains have formed in WEG ZnPc films.

First-principles density functional theory (DFT) calculations were performed to explore the electronic structure of crystalline ZnPc. The calculated band structure (Fig. 1e) reveals that α-ZnPc crystal is a direct bandgap (1.192 eV) semiconductor with band-widths of 0.318 eV and 0.057 eV for the conduction band and valence band, respectively. For a direct bandgap semiconductor, free carriers can be generated via the photo-induced interband transition, while free carriers recombination would give rise to band-edge emission^26^.

**Figure 2:**
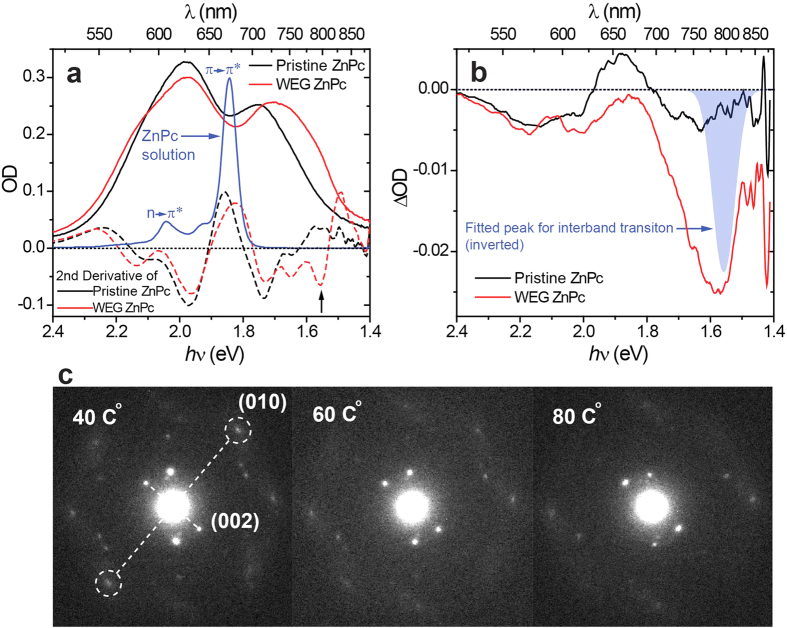
Absorption properties of ZnPc films. (**a**) UV-visible absorption spectra (solid lines) and the corresponding second derivatives (dashed lines) of pristine ZnPc (black color) and WEG ZnPc (red color) films, together with the absorption spectrum of ZnPc in DMSO (blue solid line). (**b**) Thermal-induced difference absorption spectra between 60 °C and room temperature of pristine ZnPc (black line) and WEG ZnPc (red line) films in comparison with the expected interband transition peak (blue filled peak, inverted). (**c**) Temperature-dependent SAED of a WEG ZnPc film.

Figure 2a plots the steady-state ultraviolet (UV)-visible absorption spectra of pristine ZnPc and WEG ZnPc films with their corresponding second derivatives. The absorption spectrum of ZnPc in DMSO (Dimethyl sulfoxide) is also plotted where the characteristic absorption peaks for the monomer are exhibited, i.e., a π → π* transition at 673 nm (Q band) and an n → π* transition at 607 nm, as previously assigned^27^. According to Davydov’s theory, a given molecular energy level may be split into as many components as there are inequivalent molecules per unit cell when forming molecular crystals^28^. Therefore these two ZnPc monomer absorption bands in the visible region would be split into doublets for ZnPc films. The second derivative of the absorption spectrum for pristine ZnPc films demonstrates that four Davydov splitting bands can be clearly discerned in this spectral region (see Supplementary Fig. 2 for specific assignments), while that of WEG ZnPc films develops an additional single peak at approximately 795 nm at the absorption edge (indicated by the arrow in Fig. 2a). By employing multi-component fitting of the absorption spectra, the spectrum of this specific absorption peak can be resolved (Supplementary Fig. 3), shown in Fig. 2b as an inverted blue-filled peak. Upon heating WEG ZnPc films from room temperature to 60 °C, this specific absorption band exhibits a much larger decrease than those associated with Davydov splitting (Fig. 2b). Also upon heating, the SAED pattern of WEG ZnPc films becomes more dispersed (Fig. 2c), especially those peripheral spots (lattice constant 3.61 Å derived) corresponding to the diffraction from (010) planes. These observation strongly suggests: (i) this specific absorption band is related to the long range periodicity of the crystal lattice which can easily be perturbed by a rise in temperature and (ii) the crystal lattice in WEG ZnPc films can provide enough strong intermolecular electronic coupling for a band-like electronic structure, similar to what has been observed in pentacene thin films^29^. Thus, we tentatively assign this absorption band to the predicted interband transition.

**Figure 3:**
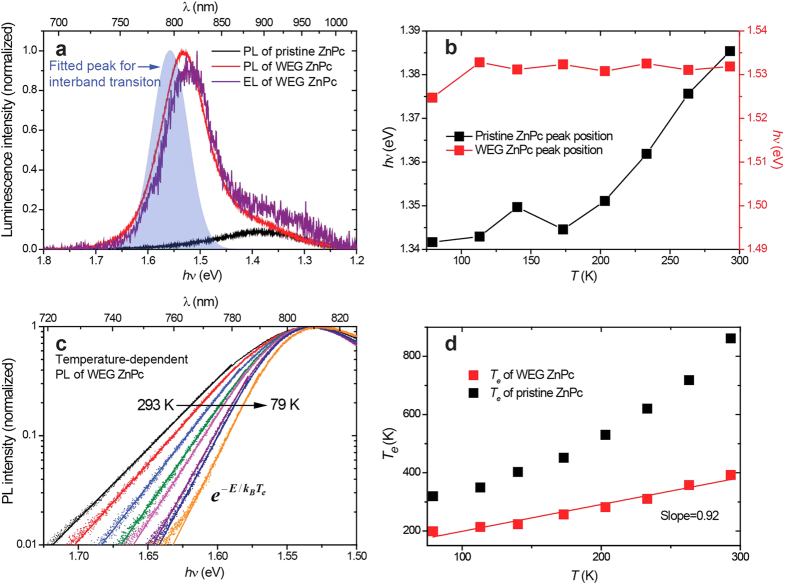
PL and EL spectra of ZnPc films excited at 532 nm and temperature-dependent behaviors of the PL. (**a**) PL spectra of pristine ZnPc (black line) and WEG ZnPc (red line) films in comparison with the EL spectrum of a WEG ZnPc film (purple line) and the assigned interband transition peak (blue filled peak). (**b**) Peak positions of the PL of pristine ZnPc (black squares) and WEG ZnPc (red squares) films under different temperatures. (**c**) Fitting the high-energy slopes of PL spectra of a WEG ZnPc film under different temperatures to Boltzmann distributions. (**d**) The fitted effective electron temperatures of pristine ZnPc (black squares) and WEG ZnPc (red squares) films. The red solid line represents a linear fitting with a slope of 0.92 and an intercept of 106.8 K.

The photoluminescence (PL) spectra of pristine ZnPc and WEG ZnPc films are plotted in Fig. 3a, along with the electroluminescence (EL) spectrum of WEG ZnPc films and the previously assigned interband transition peak for comparison. These spectra are normalized except for the PL spectrum of pristine ZnPc films which is scaled such that the intensity of the accompanying Stokes Raman scattering is equal to that of WEG ZnPc films (Supplementary Fig. 4). The high uniformity of the PL and EL spectra indicates that the photogenerated intermediates undergo the same radiative recombination process as that of the injected electrons and holes from the electrodes. The emission peaks are red-shifted by only 0.03 eV with respect to the interband transition peak, strongly suggesting that the luminescence arise from band-edge recombination of the photogenerated and electrode-injected free carriers. The PL spectrum of pristine ZnPc films is also plotted in Fig. 3a, which overlaps well with the tailing peak of the PL/EL of WEG ZnPc films. It is broader and exhibits a very low intensity (same order of magnitude as Raman scattering, Supplementary Fig. 4) and a much larger red-shift with respect to the interband transiton of WEG ZnPc films, indicating that the excitons formed in pristine ZnPc films has a charge transfer nature^5^,^30^,^31^,^32^.

Temperature-dependent PL measurements of pristine ZnPc and WEG ZnPc films were carried out to further investigate their recombination processes. The spectral shapes of both films remained essentially the same as the temperature drops, yet some peak shift and band shrink were detected.

As shown in Fig. 3b, the PL peak position dependence on the temperature reveals a remarkable difference between pristine ZnPc and WEG ZnPc films. Explicitly, the PL peak position of the pristine ZnPc film exhibits an almost monotonically blue-shift of 0.04 eV from 79 to 293 K which is similar to the behavior of CT excitons observed in blended organic conjugated polymers^33^,^34^, while that of the WEG ZnPc film stay almost unchanged. For inorganic semiconductors, the variation of the bandgap energy can be well fitted numerically by the Varshni’s expression, 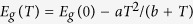, where *a* and *b* are fitting parameters^35^. At higher temperature ranges, this equation predicts a linear dependency of bandgap *E*_*g*_ on temperature *T*. Theoretical study shows that such a temperature dependency comes from a net effect of two opposite contributions, i.e., thermal expansion of the crystal lattice which results in a blue-shift of the bandgap energy and electron-phonon interaction which causes a much larger red-shift in the bandgap energy as the temperature increases^36^. For organic crystal, the interaction between neighboring molecules is of non-covalent, therefore the thermal expansion effect would be much greater than that in the inorganic semiconductors, thus contributes a much larger bandgap blue-shift. In this case, these two contributions may balance out which accounts for the observed unchanging of the PL peak position of the WEG ZnPc film with the increasing temperature. In short, these facts strongly indicate that the PL of the WEG ZnPc film is related to the band-like character of its electronic structure, while the PL of the pristine ZnPc film comes from the CT state. This indication is further supported by PL intensity dependence on the excitation intensity.

PL intensity dependence on the excitation intensity is informative for identification of the underlying recombination processes. It has been shown that the low-temperature PL intensity of the direct bandgap semiconductors relates to the excitation intensity in a power law, i.e. 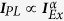, where *I*_*PL*_ is the PL intensity and *I*_*Ex*_ is the excitation intensity which is varied over a range of less than two orders of magnitude^37^,^38^. For excitation laser energies exceeding the bandgap energy, the exponent *α* is generally 1 < *α* < 2 for the free and bound-exciton emission and less than 1 for free-to-bound and donor-acceptor pair recombination. When the energy of the laser is tuned to the semiconductor bandgap energy, the exponent *α* becomes equal to 1 for the free exciton emission^38^. The free exciton mentioned here refers to a state in inorganic semiconductors formed by a pair of free electron and hole with a binding energy of the magnitude of k_B_T. As observed from the low temperature (77 K) PL peak intensity dependence on the excitation intensity of WEG ZnPc and pristine ZnPc films in logarithmic coordinates (Supplementary Fig. 5), the linear relation holds spanning more than three orders of magnitude with an *α* value of 0.85 and 0.70 for these two different samples respectively, where the excitation energy is just slightly higher than that of the bandgap energy. Therefore, we can assign the recombination process in WEG film to the free electron-to-bound exciton (with holes being more localized) recombination, and that in the pristine ZnPc films to the CT state recombination (donor-acceptor pair recombination).

As to the band shape, it has been showed that the PL spectral shapes of typical inorganic semiconductors such as degenerate GaAs, InP and InSb correspond closely to the energy distributions of electrons in the conduction band due to free-electron recombination across the bandgap. Moreover, theoretical modeling of the near-band-edge emission spectra of GaN has implied that the electron temperature is reflected in the high-energy slope of the PL and EL spectra^26^,^39^. Assuming a Boltzmann approximation for the electron distribution in the conduction band, Fig. 3c plots the fitting of the high-energy slope of the PL spectra of a WEG ZnPc film acquired at different temperatures, showing that they well-approximate the formula 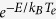, where *E* denotes the emission energy, *k*_*B*_ is the Boltzmann constant and *T*_*e*_ defines the effective temperature of free electrons. The *T*_*e*_-*T* curve (red squares in Fig. 3d) shows that the effective electron temperature obtained from the fitting is linearly correlated to the temperature of the substrate with a slope close to one unit (0.92). The intercept of 106.8 K indicates that the excess thermal energy of the hot electrons is much less than 1 *k*_*B*_*T*, meaning that the electrons are almost in a thermal equilibrium with the lattice when emission happens. Same Boltzmann fitting was also applied to temperature-dependent PL spectra of a pristine ZnPc film (Supplementary Fig. 6), however the *T*_*e*_-*T* curve it yielded distinctly deviated from any linear relation (black squares of Fig. 3d).

### Time-resolved spectroscopic characterization

**Figure 4:**
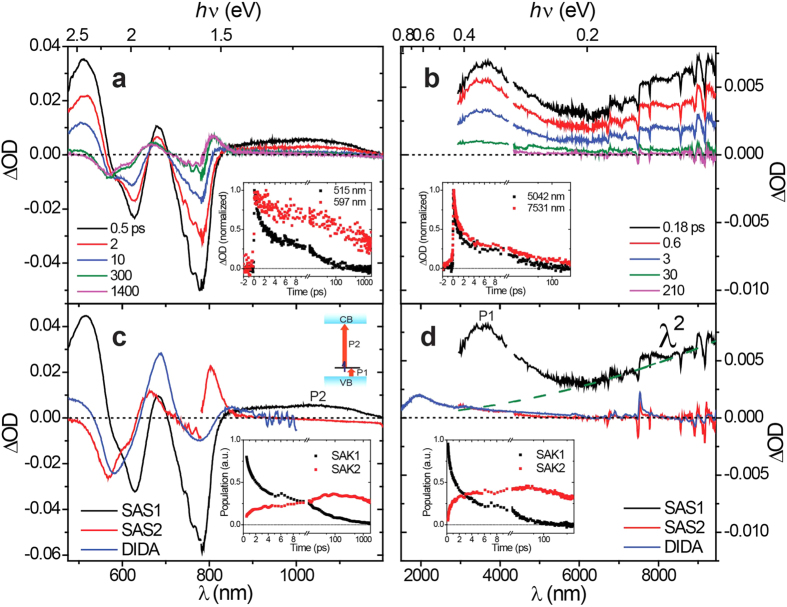
Femto-second time-resolved transient absorption spectra of WEG ZnPc films and SVD analyses resolved results. (**a,b**) As-acquired time–resolved transient absorption spectra of WEG ZnPc films in the visible-to-near-IR region and mid-IR region (the spectra in the near-IR region are multiplied by a factor of 3.8, and the spectra in the 2941 ~ 4203 nm region multiplied by a factor of 0.7 for spectral merging). Inset in (**a**): normalized kinetics probed at 515 nm (black squares) and 597 nm (red squares); inset in (**b**): normalized kinetics probed at 5042 nm (black squares) and 7531 nm (red squares). (**c,d**) SVD analyses resolved SASs (solid black and red lines) in the visible-to-near-IR and mid-IR regions with corresponding typical SAKs plotted in the insets. The solid blue lines are DIDA of a WEG ZnPc film. The dashed olive line represents the calculated curve for the free electron absorption in the form of λ^2^.

**Figure 5:**
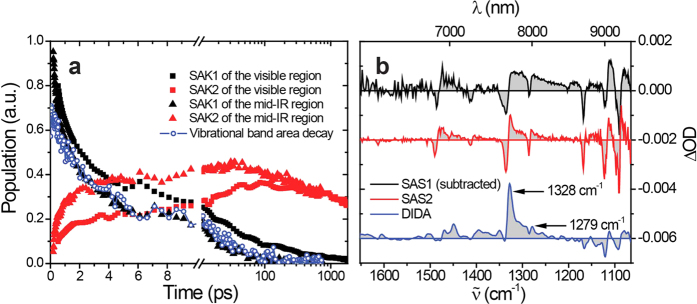
Comparison of SAKs in different spectral regions and of transient vibrational spectra. (**a**) A comparison of the SAKs obtained from SVD analyses of the visible region and mid-IR region (7352 to 9447 nm). (**b**) An expanded view of the vibrational spectral region of Fig. 4d. For clarity, the free electron absorption envelope in the form of λ^2^ has been subtracted from SAS1 and the plots are vertically displaced. The area decay for the broadened vibrational bands (blue circles) is also plotted in **(a**) for comparison with the SAKs.

To further “visualize” the photogenerated carriers and their evolution kinetics in WEG ZnPc films, ultrafast transient absorption spectroscopy spanning the visible to mid-infrared (IR) region was acquired exciting the interband transition (at 800 nm) except for that of the near-IR region (excited at 617 nm, transient absorption data of this region are basically the same as that excited at 800 nm (see Supplementary Fig. 7). For better signal-to-noise ratio, transient absorption data excited at 617 nm are presented). The transient absorption spectra in the visible-to-near IR and mid-IR regions at typical delay times are plotted in Fig. 4a,b, respectively. From the temporal evolution of the transient absorption spectra, a two-state process can be perceived. By singular value decomposition (SVD) analyses^40^,^41^,^42^,^43^, all the transient absorption spectra can be resolved into two transient components, i.e., a rapid decaying species and a rapid growing, slow decaying species. The resolved species-associated spectra (SASs) are plotted in Fig. 4c,d, with typical corresponding species-associated kinetics (SAKs) shown in the insets. The SAKs of the visible region and mid-IR region (7352 to 9447 nm) are compared in Fig. 5a. As observed, they reflect the same evolution processes, while the difference in the decay/rise phases between them can be attributed to the difference in excitation fluence since the relaxation process includes a second-order rate equation as latter discussed.

In the SAS1 (the spectra of the rapid decaying species) of the visible-to-near-IR region, two absorption peaks with apparent peak wavelengths at 518 and 683 nm exist. Such a spectral feature is consistent with that of a fully electrochemically oxidized ZnPc film that is oxidized to an extent of 1.21 electrons per ZnPc molecule, i.e., 565 nm (intense), 725 nm (moderate) and 846 nm (weak)^44^. Hence, we attribute the SAS1 in the visible-to-near-IR region to fully oxidized ZnPc cations, or holes. In the SAS1 of the mid-IR region, an apparent absorption band at approximately 3.47 μm is observed, while at longer wavelengths, a superposition of a set of transient vibrational spectra and a skewed absorption envelope extending to 9.5 μm are observed. The absorption coefficient, 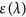, of the skewed absorption envelope rises with the increasing of wavelength as 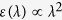 (olive dashed line in Fig. 4d), and the transient vibrational spectrum (black line in Fig. 5b) was extracted by subtraction of this *λ*^2^ absorption envelope. Such characteristic absorption envelopes have also been observed in the steady-state absorption and transient absorption spectra of typical inorganic semiconductors, where they were assigned as the absorption of free electrons^45^,^46^,^47^. According to semiconductor theory, broad IR absorption with a *λ*^*p*^ absorption coefficient-wavelength dependence is attributed to the intraband transition of free electrons and holes^48^,^49^. The value of *p* is dependent on the interaction of the electron-hole plasma with phonons and ionized impurities, as the absorption of a photon is associated with scattering of phonons or ionized impurities. In an ideal case, *p* assumes values of 3/2, 5/2, and 7/2 for scattering of acoustic phonons, optical phonons, and ionized impurities, respectively^49^. In the present case, *p* = 2, which provides direct evidence for the existence of free electrons in the conduction band. Thus, the same decaying character of SAK1 (the kinetics of the rapid decaying species) of the visible-to-near-IR and mid-IR regions represents the fast recombination process of free electrons and holes. The rising time of the mid-IR absorption envelope is within the time resolution of our experiments (~100 fs), indicating an ultrafast generation of free carriers.

**Figure 6:**
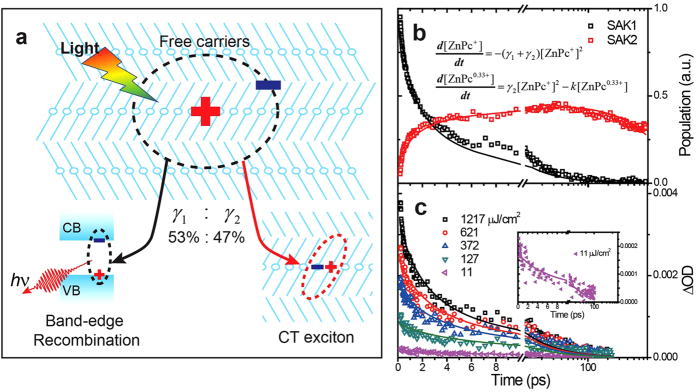
Kinetic model of light induced processes. (**a**) A schematic diagram illustrating photogeneration of free carriers and their corresponding recombination paths. (**b**) Typical SAKs were fit with the model illustrated in (**a**) and described within the text. The differential equations applied are shown inside the graph and the fitting yields *γ*_1_ = 0.46, *γ*_2_ = 0.41 and *k* = 0.67 ns^−1^. (**c**) The kinetics acquired at 7305 nm, where the signal of free electrons prevails, under different excitation fluences. The solid lines present numerical fits of the kinetics using the first differential equation in **(b**). Inset is an enlargement of the kinetics acquired with 11 μJ/cm^2^ (4.4 × 10^13^ photon/cm^2^) excitation fluence.

The assignment of SAS2 (the spectra of the rapid growing, slow decaying species) was accomplished by comparison to the iodine doping-induced difference absorption (DIDA) spectra of a WEG ZnPc film (blue solid line in Fig. 4c,d). The DIDA spectra were obtained by exposing a WEG ZnPc film to I_2_ vapor (see Supplementary Information including Supplementary Fig. 8). Iodine only partially oxidizes metal phthalocyanines in the solid phase to form a π-cation radical species with a nominal charge of about +0.33 rather than a full unit^50^. This behavior has been referred to as “partial oxidation”, “mixed valence” or “incomplete charge transfer”^51^. The good correspondence between the SAS2 and DIDA spectra led us to assign SAS2 to partially oxidized ZnPc cations that possess a nominal charge +δ (ZnPc^δ+^, 0 < δ < 1), referred to as CT excitons. The correlation between the decay of SAK1 and the rise of SAK2 (the kinetics of the rapid growing, slow decaying species) strongly indicates that the CT excitons are produced from the localization of free electrons and holes. This localization process is depicted in Fig. 6a.

Figure 5b presents an expanded view of Fig. 4d comparing the vibrational spectral region. All the vibrational bleaching peaks match well, in accordance with the Fourier transform infrared (FTIR) spectrum of ZnPc layers^52^. Notably, the absorption peaks at 1328 and 1279 cm^−1^ of SAS2 are comparable to the DIDA spectrum, consistent with the previous CT exciton assignment, while those for SAS1 are much broader. To evaluate the decay kinetics of this broad vibrational band, the amount of area between 1316 and 1294 cm^−1^ where the contribution of the localized CT excitons is comparatively small, is calculated from the transient absorption spectra (the free electron absorption contributions in forms of λ^2^ were subtracted) and the evolution kinetic of the amount of area (blue circles) is plotted in Fig. 5a after the baseline correction. As observed, this evolution curve almost coincides with SAK1 of the mid-IR region, which indicates that the broadening of the molecular vibrational peaks is due to the formation of free carriers. It has been shown that the vibrational peaks of conjugated polymers can be modulated by charges (cations and anions) to exhibit intense bands, and it was further proposed that the intensity of the IR bands can be diagnostic for bound ion pairs and their escape to form free ions^53^. By analogy, we propose that the shrinking of the vibrational bands observed in our case corresponds to the localization of the delocalized free carriers to the CT excitons.

Until now, there remain two absorption bands, centered at 1054 nm (1.176 eV) and 3.47 μm (0.357 eV) in SAS1, to be assigned. The summation of these two transition energies (1.533 eV) is almost identical to that of the interband transition energy (1.558 eV), suggesting that these two transitions may involve a mid-gap energy level comparable to that of the polaron level (cation) in conjugated polymers^54^,^55^, as depicted in the energy diagram in Fig. 4c. We also observed that the line-shape of the absorption band at 3.47 μm is quite similar to the fingerprint absorption band of the I_2_-oxidized ZnPc radical cation centered at 1.93 μm (0.644 eV). So we tentatively assign the 1.93 μm (0.644 eV) absorption band in DIDA and the 3.47 μm (0.357 eV) absorption band in SAS1 to P1 transitions of the ZnPc^δ+^ cation and ZnPc^1+^ cation (trapped hole) respectively, where the P1 transition denotes the transition of an electron from the valence band to the mid-gap energy level attributed to the cations. The P1 transition energy of ZnPc^1+^ is smaller than that of ZnPc^δ+^ because the electron involved in the transition does not encounter a Columbic repulsion from the localized negative charge in the CT exciton. Such repulsion energy would be identical to the binding energy of the CT exciton in magnitude, but opposite in sign. Thus, the energy difference (0.287 eV) can be used to estimate the charge separation distance of the CT exciton using Coulomb’s law employing a point charge approximation. Assuming 3.4 for the dielectric constant^56^, the charge separation distance of the CT exciton was calculated to be 1.48 nm, comparable to the dimension of one molecule. Accordingly, the absorption band centered at 1054 nm (1.176 eV) in SAS1 is assigned to the transition of the unpaired electron in the ZnPc^+1^ cation to the conduction band, denoted as P2 in the energy diagram in Fig. 4c. For the ZnPc^δ+^ cation, the P2 transition band appears at 852 nm (1.455 eV), giving a Columbic interaction of 0.279 eV, which is consistent with that calculated from P1 transitions.

The schematic diagram in Fig. 6a summarizes the main photoinduced physical processes in WEG ZnPc films, i.e., the interband photoexcitation leads to the generation of delocalized free carriers that undergo relaxation processes involving: (i) band-edge recombination generating photo-emissions, and (ii) charge localization to form CT excitons. These processes can be accounted by the second-order rate equations as written in Fig. 6b. The first equation describes the relaxation of free carriers where *γ*_1_ and *γ*_2_ denote the second-order rates of electron-hole recombination and localization, respectively. The second equation describes the forming and decaying of CT excitons where *k* denotes the decay rate constant. Typical SAKs were fit to the second-order rate equations, which gives a branch ratio of *γ*_1_:*γ*_2_ that is close to 1:1 and an exponentially decay lifetime of 1.48 ns for CT excitons.

Based on the mobility and lifetime of free carriers, the diffusion length can be calculated by incorporating the following two equations, 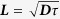 and 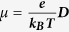
57,58, where *L* is the diffusion length, *μ* denotes the carrier mobility, *D* represents the diffusion coefficient, τ defines the carrier lifetime and *e* is the elementary charge. Since the mobility of the photogenerated short-lived hot carriers could be much higher than that of thermalized carriers (which is not available), we use the static hole mobility of phthalocyanine single-crystal transistors^59^ *μ*_***h***_ = 1 cm^2^ V^−1^s^−1^ along the stacking axis in the calculation to estimate a lower limit of the hole diffusion length. Taking *τ* = 6.8 ps as the hole lifetime which was estimated from the time required for the electron transient absorption to decay to 1/e of its maximum under the lowest excitation fluence used (Fig. 6c), we obtain a lower limit of the diffusion length for holes of 4.2 nm, which corresponds to a distance of 11 ZnPc molecules along the stacking axis.

## Conclusion

We have shown the existence of photogenerated free carriers in highly crystalline WEG ZnPc films, although their lifetime is only several ps. This behavior is proposed to arise from the effective intermolecular interactions among the cofacially-stacked π-conjugated ZnPc molecules achieved through tight and highly ordered packing. Especially, a small reduction in the intermolecular π-π stacking distance would effectively turn the photoexcitation of organic semiconductors from the exciton to band-like electronic nature^60^, and significantly improve the carrier transport properties which has been illustrated in other organic semiconductor films of 6,13-bis(triisopropylsilylethynyl) pentacene, where the hole mobility was increased almost by a factor of 6 when the π-π stacking distance was altered from 3.33 Å to 3.08 Å^25^. Our result also will stimulate new possibilities for the fabrication and design of organic semiconductor-based devices in parallel to inorganic semiconductors in views of interband transitions.

## Additional Information

**How to cite this article**: He, X. *et al.* Photogenerated Intrinsic Free Carriers in Small-molecule Organic Semiconductors Visualized by Ultrafast Spectroscopy. *Sci. Rep.* **5**, 17076; doi: 10.1038/srep17076 (2015).

## Supplementary Material

Supplementary Information
