# Supplementary material for: Photogenerated Intrinsic Free Carriers in Small-molecule Organic Semiconductors Visualized by Ultrafast Spectroscopy
Source: Sci Rep. 2015 Nov 27;5:17076. doi: 10.1038/srep17076 (PMC4661463; doi:10.1038/srep17076)
Supplement: Supplementary Information [file srep17076-s1.pdf]

Photogenerated Intrinsic Free Carriers in Small-molecule Organic Semiconductors Visualized by Ultrafast Spectroscopy

Xiaochuan He[1], Gangbei Zhu[1], Jianbing Yang[2], Hao Chang[2], Qingyu Meng[3], Hongwu Zhao[3], Xin Zhou[4], Shuai Yue[1], Zhuan Wang[1], Jinan Shi[5], Lin Gu[5], Donghang Yan[2]*, Yuxiang Weng[1]*

[1]Laboratory of Soft Matter Physics, Institute of Physics, Chinese Academy of Sciences (CAS), Beijing 100190, China.

[2]State Key Laboratory of Polymer Physics and Chemistry, Changchun Institute of Applied Chemistry, CAS, Changchun 130022, China.

[3]Laboratory of Solid State Quantum Information and Quantum Computation, Institute of Physics, CAS, Beijing 100190, China.

[4]State Key Laboratory of Catalysis, Dalian Institute of Chemical Physics, CAS, Dalian 116023, China.

[5]Laboratory of Advanced Materials & Electron Microscopy, Institute of Physics, CAS, Beijing 100190, China.

*e-mail: yxweng@aphy.iphy.ac.cn; yandh@ciac.ac.cn

Sample preparation. ZnPc was purchased from Sigma-Aldrich Co. and F_{16}CuPc from Luminescence Technology Co. The inducing layer material 2,5-bis(4-biphenylyl)-bithiophene (BP2T) was synthesized with a previously described method[1]. All materials were purified twice prior to use by thermal-gradient train sublimation.

Fused quartz substrates were ultrasonicated sequentially in a NaOH solution, deionized water and alcohol for 30 min in each process, and then dried at 120 °C for 30 min in an oven to eliminate the influence of moisture. During weak epitaxy growth (WEG) of ZnPc films, a 5 nm layer of BP2T was thermally evaporated on fused quartz substrates at a substrate temperature of 155 °C. A 40 nm layer of ZnPc was then deposited onto the BP2T film with a substrate temperature maintained at 150 °C. All the organic thin films were deposited in a vacuum chamber at 10^{-4}-10^{-5} Pa and the deposition rate was about 1 nm/min. Film thicknesses were monitored by a quartz-crystal microbalance during deposition. The fabrication condition for pristine ZnPc films was identical to that for WEG ZnPc films, except for the absence of the BP2T inducing layer.

For the samples measured in the mid-infrared (IR) region, we used 0.5 mm thick CaF_2 plates as replacement substrates for fused quartz, which were cleaned with acetone and alcohol.

For the sample prepared for electroluminescence (EL) measurements, indium-tin oxide (ITO)-coated glass with a sheet resistance of 15 Ω/□ was used as the substrate.

Before deposition, the substrates were cleaned with detergent, and then ultrasonicated sequentially in acetone, alcohol and deionized water and dried in pure N_2. Subsequently, poly(3,4-ethylenedioxythiophene):poly(styrenesulphonate) (PEDOT:PSS) (H.C.Starck P VP Al 4083) was spin-coated on top of the substrate at 3000 rpm for 30 s to smooth the interface and then dried at 150 °C for 15 min under the ambient condition. After deposition of BP2T and ZnPc, a 1 nm layer of F_{16}CuPc was deposited on the ZnPc film as a buffer layer at temperature of 150 °C. Finally, the sample was transferred rapidly to another vacuum chamber for deposition of an Au layer of about 60 nm thick, which served as the electrode, by thermal evaporation with a shadow mask at 10^{-4} Pa.

Morphology characterization. The AFM imaging of pristine ZnPc and WEG ZnPc films were carried out by a SPI 3800/SPA 300HV (Seiko Instruments Inc., Japan) with tapping mode.

To acquire SAED of WEG ZnPc films, a 40-nm thick ZnPc film was vacuum deposited on 5-nm thick amorphous SiN using WEG method. The WEG ZnPc film was mounted on a holder with heating function and the temperature-dependent SAED was measured by a TEM with a thermionic gun (JEOL, JEM 2010). During the measurement, the intensities of electron beams were controlled as low as possible (spotsize: 5, current density: ~0.0 pA/cm^2) to minimize possible radiation damage to the sample.

Spectroscopic characterization. Ultraviolet (UV)-visible absorption spectra of the ZnPc films were measured with a U-3010 spectrophotometer (Hitachi). When the temperature-dependent UV-visible difference spectra were measured, the films were attached to a metallic water jacket as the sample holder, and the temperature was controlled with a circulating water bath (HAAKE DC30-K20, Thermo Scientific). Steady-state IR absorption spectra were measured in vacuum using a Fourier transform infrared (FTIR) spectrometer (VERTEX 70v, Bruker). Each FTIR spectrum was recorded at a resolution of 4 cm^{-1} and 20 scans were signal-averaged.

Photoluminescence (PL) spectra of the ZnPc films were acquired on a Raman microscopic spectrometer (LabRAM HR800, HORIBA Jobin Yvon) in PL configuration due to the extremely low photoluminescence intensity. A cryostat (TMS 94, Linkam Scientific Instruments) was used to control the temperature of the sample when the temperature-dependent PL spectra were measured. The excitations used were 532 nm and 785 nm lasers, and the size of the excitation spot on the sample was about 1 μm.

For EL measurements, the bottom negative electrode (ITO) and top positive electrode (Au) were connected to a source meter (Keithley 2601A) using soft Au probes. The sample was first subjected to a 0 V~2.5 V~0 V process. Then a durative -3 V voltage was applied to the sample. The EL spectra of the sample were collected with a low-noise CCD (Princeton Instrument ProEM: 512B) coupled spectrometer (Princeton Instrument Acton 2300) with an integration time of 1s.

To obtain standard spectral fingerprints for cations in the WEG ZnPc film, a sample with CaF_2 as the substrate was exposed to I_2 vapor for 30 s to produce ZnPc cations by oxidation with I_2. UV-visible and FTIR spectra were measured after and before I_2 exposure from which the doping-induced difference absorption spectra in the visible region and IR regions were derived.

Time-resolved transient absorption measurements. (i) In the visible region. Transient absorption spectra in the visible region were measured with a the pump-probe setup as described elsewhere[2]. Briefly, the fundamental output of a pulsed laser with a central wavelength of 800 nm, a pulse duration of 150 fs (FWHM, full-width at half-maximum) and a repetition rate of 1 kHz from an amplified Ti:Sapphire laser (Hurricane, Spectra Physics) was split into two beams by an optical wedge. The more intense beam passed through a delay line and was focused onto the sample as the pump beam, while the weaker one was focused into a sapphire plate to generate a white light continuum as the probe beam. A filter was used to remove the 800 nm residual in the white light continuum, which was then split into two probe beams denoted as the signal and reference beam by a 1:1 broad-band beam splitter. The signal beam was focused at the same spot as the pump on the sample. The polarization between the pump and probe was set to the magic angle (54.7 °) with an 800 nm half-wave plate. Reference light and transmitted signal light were collected by optical fibers and sent through an imaging spectrograph (SpectraPro-150,

Acton Research) to be acquired by a CCD (128HB, Acton Research). The as-acquired data were further smoothed, and corrected of the chirp effect using the chirped white light continuum as a reference, which was measured by the optical Kerr gating effect employing a quartz plate as the Kerr medium. The excitation wavelength was 800 nm for both pristine ZnPc and WEG ZnPc films, with excitation energies of ~400 μJcm^{-2}/pulse and ~300 μJcm^{-2}/pulse, respectively. Both measurements were conducted in the ambient environment.

(ii) In the near IR region. The experimental setup for measuring transient absorption spectra in the near-IR region was different to that used for the visible region. Briefly, the fundamental output of a pulsed laser with a central wavelength of 800 nm, a pulse duration of 120 fs (FWHM) and a repetition rate of 333 kHz from an amplified Ti:Sapphire laser (Spitfire, Spectra Physics) was split into two beams. One beam passed into an OPA (OPA-800 CF-1, Spectra Physics) to generate the excitation light while the other beam was focused into a sapphire plate to generate white light continuum as the probe beam. The polarization between the pump and probe was set to the magic angle (54.7 °) and both were focused onto the sample at the same spot. Before focusing, the probe beam was filtered by a long-pass filter so that only the near-IR light could pass. The excitation beam traveled through a delay line and was modulated by a mechanical chopper at a frequency of half of the laser repetition rate. The transmitted probe light was collected by an optical fiber and sent through an imaging spectrograph (SpectraPro 2300i,

Princeton Instruments) to be acquired by an InGaAs array detector (OMA-V, Princeton Instruments). For both pristine ZnPc and WEG ZnPc films, the excitation wavelength was 617 nm and the excitation energy was ~50 μJcm^{-2}/pulse. The measurements were conducted in the ambient environment.

(iii) In the mid-IR region. Transient absorption spectra in mid-IR region were acquired with the following experimental setup. The output of an 800 nm centered pulsed laser with a duration of 35 fs (FWHM) at a repetition rate of 1 kHz from an amplified Ti:Sapphire laser (Spitfire Ace, Spectra Physics) was split into two beams by a 800 nm beam splitter. One of the beams passed through a delay line and was modulated by a mechanical chopper at a frequency of half of the laser repetition rate, which was employed as the excitation beam. The other beam was used to generate a broadband mid-IR source as the mid-IR probe via four-wave mixing through filamentation in air[3]. During this process, the fundamental beam was compressed with a telescope and sent through a beta barium borate (BBO) crystal to generate the second harmonic generation (SHG). After SHG, the fundamental and second harmonic beams were separated with a dichroic mirror for individual adjustment of their relative time delays and polarization. The fundamental beam first passed through a short delay line such that its time delay could be finely adjusted, then passed through a half-wave plate that rotated its light polarization by 90 °, and finally recombined with the second harmonic beam by another dichroic mirror. The recombined beams were focused simultaneously by an aluminum

concave mirror into the air, where a bright filament formed, and a broad-band IR light was generated through the nonlinear optical processes in the filament[3]. A black screen was set some distance behind the filament, which was vertically adjusted such that it blocked most of the visible light while allowing enough mid-IR light as the probe to pass over the top edge of the block. Another aluminum concave mirror collected the mid-IR light and focused it onto the sample. Transmitted mid-IR light was collected, sent into an imaging spectrometer (iHR 320, HORIBA Jobin Yvon) and acquired by a 64-channel MCT array detector of Femtosecond Pulse Acquisition System (FPAS-0144, Infrared Systems Development). Both the spectral coverage and the peak wavelength of the IR probe generated from the filament are sensitive to the optical alignment of the mid-IR generation. To optimize the signal-to-noise ratio, we divided the spectrum range from 2941 to 9447 nm into three spectral segments for independent measurement by tuning the peak wavelength into the three spectral windows individually: from 2941 to 4202 nm, 3701 to 8298 nm (CO_2 absorption interference occurring in this range) and 7352 to 9447 nm, with a partial spectral overlap between the adjacent segments. Due to the limited detection range of the spectrometer, three spectral segments were further divided into slices for spectral acquisition. Finally, the full spectrum was obtained by merging all the individually measured spectral slices, where the overlapped spectral regions between two adjacent slices were used for data scaling. The excitation wavelength was 800 nm and

with an excitation energy of ~1200 μJ cm^{-2}/pulse. The measurements were conducted in the ambient environment.

Band structure calculation. WEG ZnPc films have been characterized to be in the metastable α-phase (monoclinic, space group C2/n, which is referred to as γ-phase in some recent papers since α-phase was redetermined to be of a monoclinic P -1 space group crystal structure[4]) by X-ray diffraction (XRD) and selected area electron diffraction (SAED)[5] analyses. Because no detailed structural data of α-ZnPc is available, generally the structural data of isomorphic CuPc[6] is used as the first approximation. This approach has been previously applied in calculating XRD patterns of ZnPc films[7]. Specifically, the Cu atom in α-CuPc is replaced by a Zn atom, and the positions of the other atoms in the unit cell remain the same as those in α-CuPc. The crystal structure of an α-ZnPc primitive cell is shown in Supplementary Fig. 1a. The band structure of the α-ZnPc crystal was calculated using density functional theory (DFT) in $DMol^3$ program[8]. We performed an all-electron calculation with a double numeric polarized (DNP) basis set[8], generalized gradient approximation (GGA)[9] and the Becke-Lee-Yang-Parr (BLYP) exchange-correlation potential[10]. The k-point mesh was 4×4×1, and a Brillouin zone path was generated in the Monkhorst-Pack scheme[11] with 0.015 $Å^{-1}$ spacing as shown in Supplementary Fig. 1b.

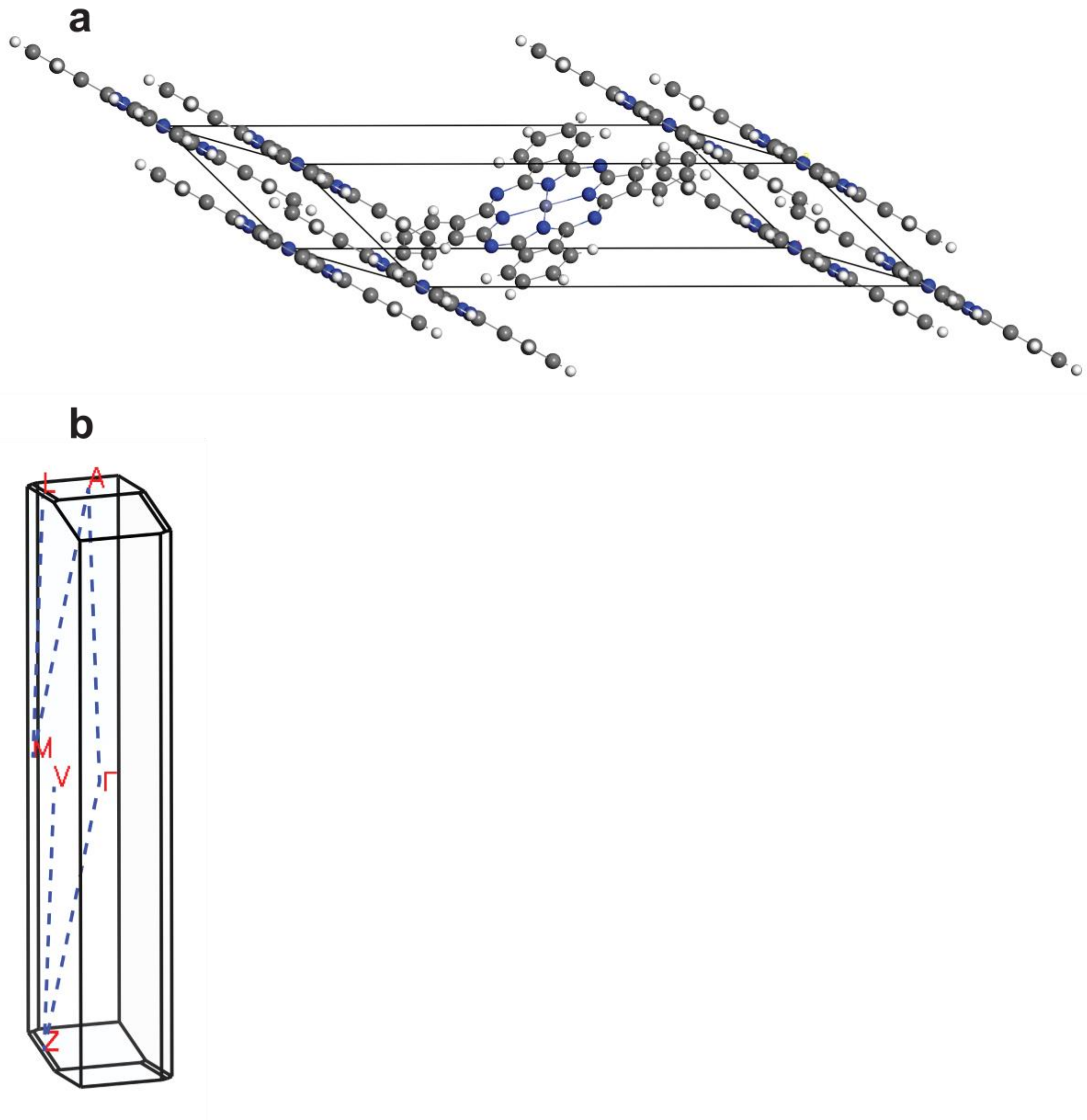

Supplementary Figure 1 | The crystal structure and reciprocal lattice of a primitive cell of the α-ZnPc crystal. (a) Illustration of a primitive cell of the α-ZnPc crystal. (b) The Brillouin zone path generated in the Monkhorst-Pack scheme shown in the reciprocal lattice.

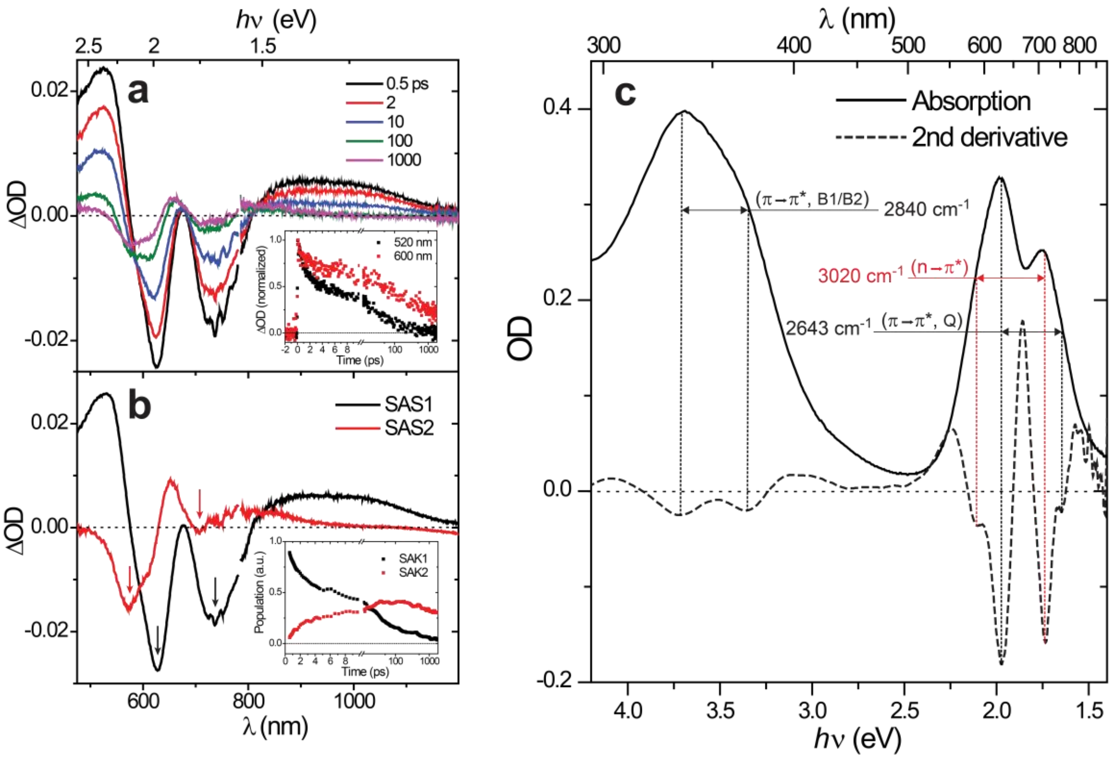

Supplementary Figure 2 | Assignment of Davydov splitting bands of pristine ZnPc films. Time-resolved transient absorption spectra of pristine ZnPc films in the visible-to-near-IR region (spectra in near-IR region are multiplied by a factor of 4 for spectral merging) are shown in (a). The inset displays the evolution kinetics probed at 520 nm and 600 nm. The two SVD resolved SASs are shown in (b), with corresponding SAKs shown in the insets, where the pair of Davydov splitting would possess identical evolution kinetics. The UV-visible absorption spectrum of pristine ZnPc films is plotted in (c), where the dashed curve represents the corresponding second derivative spectrum. The specific Davydov splitting bands are assigned by referring to both the secondary derivative spectrum and the SVD resolved SASs. The splitting energies and their

corresponding original transitions are indicated in (c) with different colors. The splitting energies obtained here are similar to those reported for α-phase CuPc determined by magnetic circular dichroism spectroscopy[12].

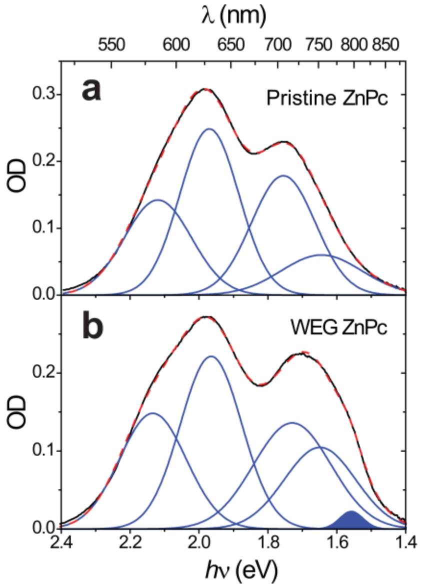

c

Pristine ZnPc Gaussian peaks	Pristine ZnPc 2nd derivative negative peaks	WEG ZnPc Gaussian peaks	WEG ZnPc 2nd derivative negative peaks
		1.55759	1.556
1.64435	1.649	1.64873	1.649
1.755	1.734	1.72921	1.729
1.97	1.971	1.96488	1.965
2.11939	2.112	2.13398	2.138

The unit is eV for all the values.

Supplementary Figure 3 | Multiple-component fitting of the visible absorption spectra of pristine ZnPc and WEG ZnPc films. The visible region absorption spectra of pristine ZnPc (a) and WEG ZnPc (b) films were fit with Gaussian peaks (blue solid lines) after linear baseline correction. The fitting results are shown as dashed red lines, and the shaded area represents the expected interband transition peak. During the fittings, the negative peak positions in the second derivative spectra were used as the guidance for presetting the corresponding absorption peak positions, and the fitted Gaussian peak values are compared with those from the second derivative spectra in (c).

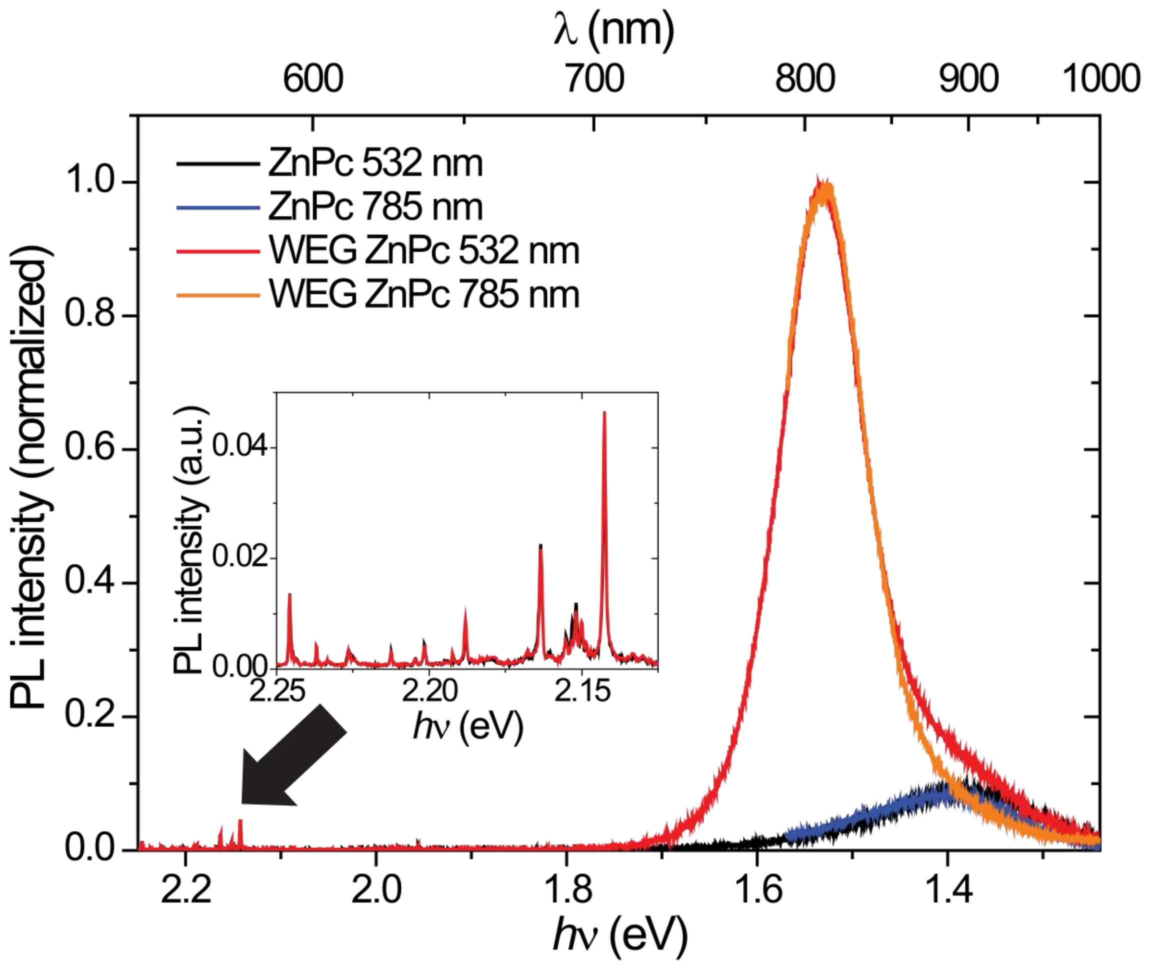

Supplementary Figure 4 | Comparison of the PL spectra excited at 532 nm with that excited at 785 nm of pristine ZnPc and WEG ZnPc films. The PL spectrum of WEG ZnPc films excited at 532 nm (red line) is normalized, and the PL spectrum of pristine ZnPc films excited at 532 nm (black line) is scaled such that the intensity of the accompanying Stokes Raman scattering is equal to that of WEG ZnPc films excited at 532 nm. The PL spectra of WEG ZnPc films (yellow line) and pristine ZnPc film (blue line) excited at 785 nm are scaled to the same level as the PL spectra of the corresponding films excited at 532 nm. As observed, the main peak of the PL spectrum of

WEG ZnPc excited at 532 nm is identical to that excited at 785 nm, indicating a fast relaxation of the free carriers to the band-edge before recombination. The inset gives an enlarged view of the Stokes Raman scatterings (denoted by a thick arrow).

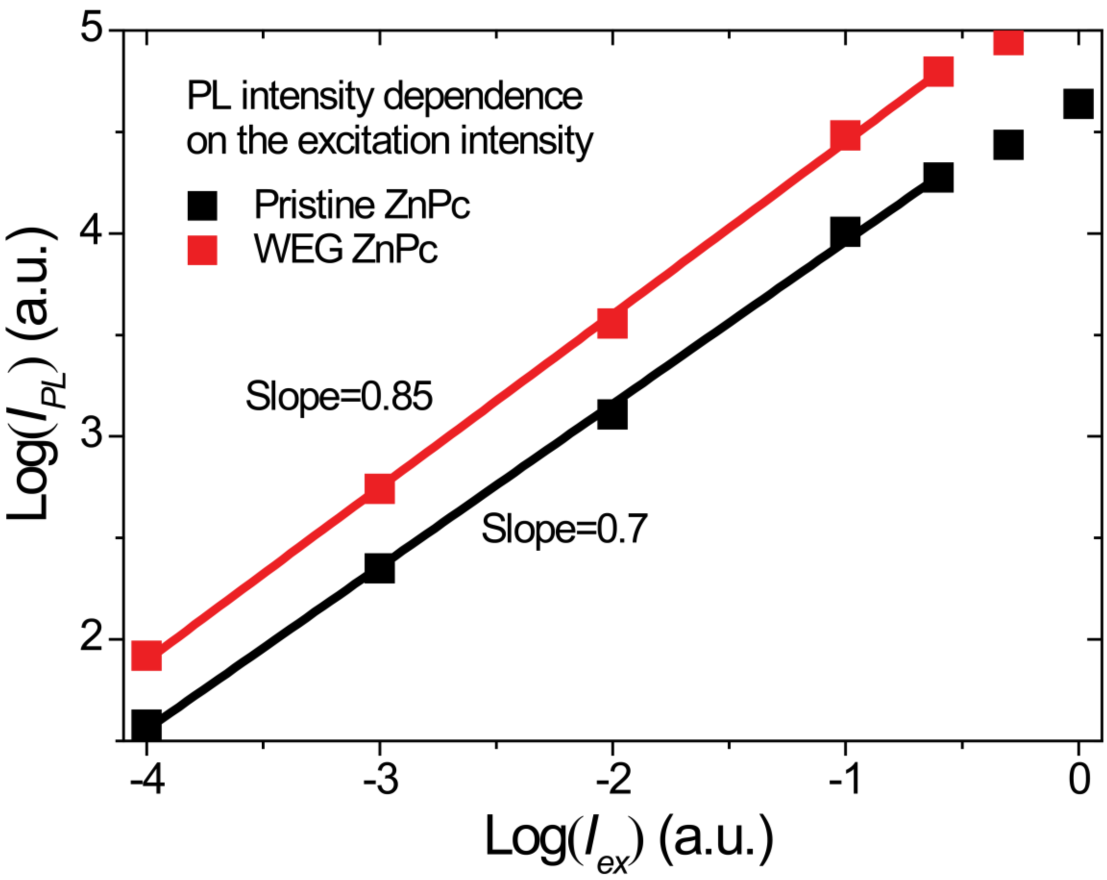

Supplementary Figure 5 | PL intensity dependence on excitation intensity of pristine ZnPc and WEG ZnPc films excited at 785 nm and measured at 77 K.

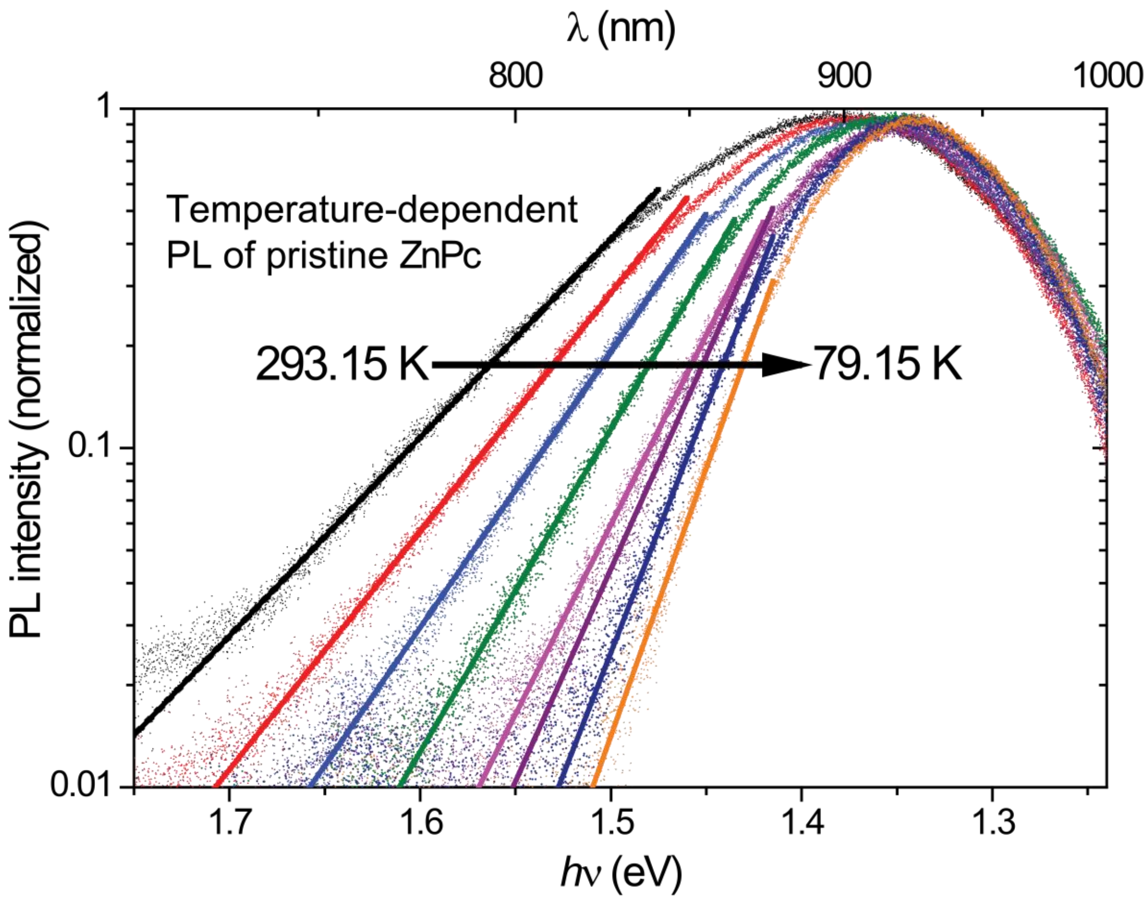

Supplementary Figure 6 | Normalized temperature-dependent PL spectra of a pristine ZnPc film (color dots) excited at 532 nm and fittings of the high-energy slopes to Boltzmann distributions (color lines).

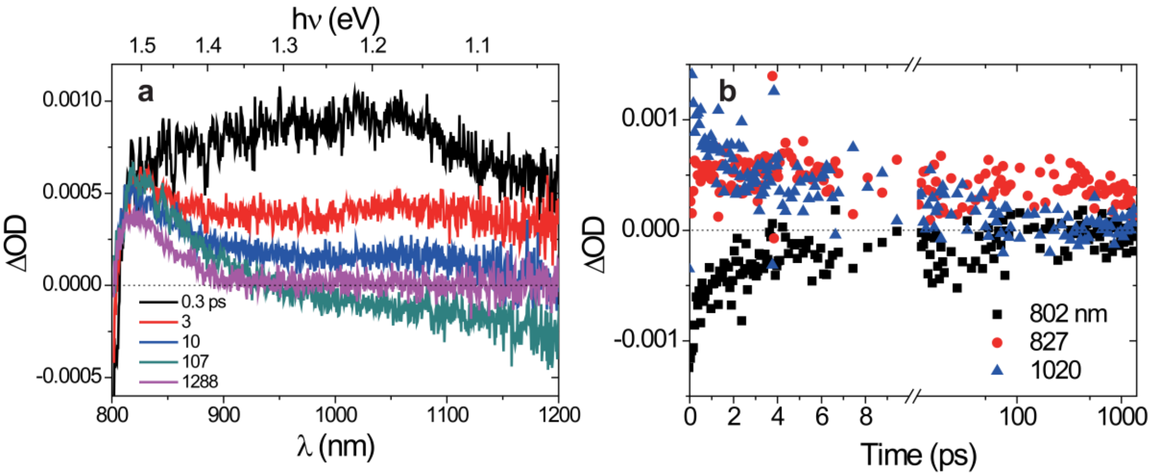

Supplementary Figure 7 | Near-IR transient absorption data of WEG ZnPc films excited at 800 nm. The spectral (a) and temporal (b) behavior is basically the same as that excited at 617 nm, however the signal amplitude and signal-to-noise ratio is much smaller than that excited at 617 nm due to a much lower excitation intensity (~50 μJ/cm^2).

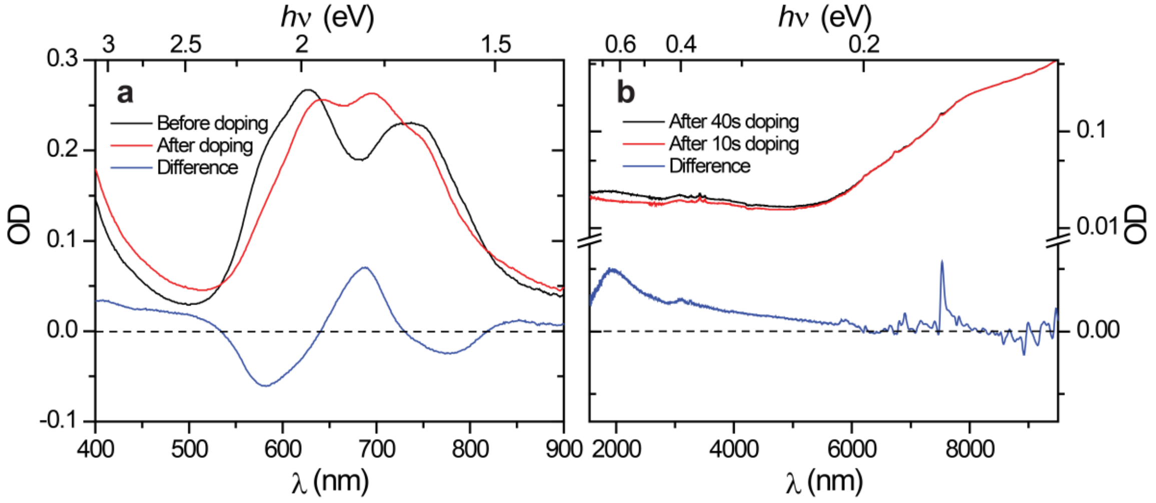

Supplementary Figure 8 | Doping-induced difference absorption spectra of a WEG ZnPc film. UV-visible (a) and FTIR (b) spectra of a WEG ZnPc film measured after (red lines) and before (black lines) I_2 exposure and the corresponding difference absorption spectra derived (blue lines).

References

1. Hotta, S., Kimura, H., Lee, S. A. & Tamaki, T. Synthesis of thiophene/phenylene co-oligomers. II [1]. Block and alternating co-oligomers. *J. Heterocycl. Chem.* **37,** 281–286 (2000).

2. Zhang, L., Yang, J., Wang, L., Yang, G. & Weng, Y. Direct Observation of Interfacial Charge Recombination to the Excited-Triplet State in All-trans-Retinoic Acid Sensitized TiO2 Nanoparticles by Femtosecond Time-Resolved Difference Absorption Spectroscopy. J. Phys. Chem. B 107, 13688–13697 (2003).

3. Fuji, T. & Suzuki, T. Generation of sub-two-cycle mid-infrared pulses by four-wave mixing through filamentation in air. *Opt. Lett.* **32,** 3330 (2007).

4. Hoshino, A., Takenaka, Y. & Miyaji, H. Redetermination of the crystal structure of α-copper phthalocyanine grown on KCl. *Acta Crystallogr. Sect. B Struct. Sci.* **59,** 393–403 (2003).

5. Wang, H., Zhu, F., Yang, J., Geng, Y. & Yan, D. Weak Epitaxy Growth Affording High-Mobility Thin Films of Disk-Like Organic Semiconductors. Adv. Mater. 19, 2168–2171 (2007).

6. Erk, P., Hengelsberg, H., Haddow, M. F. & van Gelder, R. The innovative momentum of crystal engineering. *CrystEngComm* **6,** 474 (2004).

7. Schünemann, C. *et al.* Zinc phthalocyanine — Influence of substrate temperature, film thickness, and kind of substrate on the morphology. *Thin Solid Films* **519,** 3939–3945 (2011).

8. Delley, B. From molecules to solids with the DMol 3 approach. *J. Chem. Phys.* **113,** 7756 (2000).

9. Perdew, J., Burke, K. & Ernzerhof, M. Generalized Gradient Approximation Made Simple. *Phys. Rev. Lett.* **77,** 3865–3868 (1996).

10. Miehlich, B., Savin, A., Stoll, H. & Preuss, H. Results obtained with the correlation energy density functionals of becke and Lee, Yang and Parr. *Chem. Phys. Lett.* **157,** 200–206 (1989).

11. Monkhorst, H. & Pack, J. Special points for Brillouin-zone integrations. *Phys. Rev. B* **13,** 5188–5192 (1976).

12. Hollebone, B. & Stillman, M. Assignment of absorption and magnetic circular dichroism spectra of solid, α phase metallophthalocyanines. *J. Chem. Soc. Trans. II* **74,** 2107–2127 (1978).
